# Genomic and Drug Resistance Profile of Bovine Multidrug-Resistant *Escherichia coli* Isolated in Kazakhstan

**DOI:** 10.3390/pathogens14010090

**Published:** 2025-01-17

**Authors:** Pavel G. Alexyuk, Andrey P. Bogoyavlenskiy, Yergali S. Moldakhanov, Kuralay S. Akanova, Adolat N. Manakbayeva, Timur Kerimov, Vladimir E. Berezin, Madina S. Alexyuk

**Affiliations:** Research and Production Center for Microbiology and Virology, Almaty 050010, Kazakhstan; alpagen@live.com (P.G.A.); anpav_63@mail.ru (A.P.B.);

**Keywords:** *Escherichia coli*, multidrug resistance, colibacillosis, draft genome, Illumina sequencing

## Abstract

While studying the prevalence and profile of antibiotic resistance among *E. coli* isolated from the feces of calves with signs of colibacillosis, a strain with a wide spectrum of drug resistance was isolated. Whole-genome sequencing, followed by bioinformatic processing and the annotation of genes of this strain, showed that the genome has a total length of 4,803,482 bp and contains 4986 genes, including 122 RNA genes. A total of 31% of the genes are functionally significant and represent 26 functional groups. Additionally, 55 antibiotic resistance genes were revealed. A phenotypic drug-resistance study was performed according to CASFM and CLSI guidelines, which showed that the investigated strain was resistant to eight antibacterial drugs of different classes, including colistin. This is the first report on the AMR profile of an *E. coli* isolate obtained from a sick calf with evidence of escherichiosis in Kazakhstan. The provided information on the genome will be valuable in studying the evolution and development of antibiotic-resistant forms of *E. coli* and increase our knowledge of pathogenicity. It may also be a source for future comparative studies of the virulence and drug resistance of *E. coli* isolates.

## 1. Introduction

*E. coli* is a type of commensal Gram-negative bacterium and is an integral part of the intestinal microbiota of various animals, including humans. However, some strains of *E. coli* can cause different diseases of varying severity in both humans and animals. In addition to causing direct harm to human health, pathogenic *E. coli* infections can cause significant economic losses during mass epizootics of colibacillosis among farm animals. Diarrhea and coli septicemia caused by pathogenic *E. coli* significantly reduce body weight gain in newborn calves leading to a decrease in the volume, deterioration in the quality, and increase in the cost of final products [1,2]. Despite numerous existing studies on the etiologic role of *E. coli* in the development of colibacillosis in calves and the introduction of mass treatment and preventive measures, this disease remains relevant in view of the birth rate of calves with low natural resistance. The postnatal treatment of escherichiosis mostly involves the use of antibiotic drugs [3]. However, the global spread of antibiotic resistance has significantly compromised the effectiveness of this therapeutic approach. In addition, because *E. coli* can serve as a genetic reservoir encoding mechanisms of antibiotic resistance, the entry of such strains into different habitats may spread multidrug resistance among pathogens, including pathogens dangerous to human health [4]. In vitro data indicate the potential virulence and antibiotic resistance factors to be transferred through mobile genetic elements between *E. coli* and other bacteria, primarily enterobacteria such as *Salmonella*, *Shigella,* and *Klebsiella* [5]. Analyzing antibiotic resistance in bacterial isolates obtained from various sources, including farm animals, can help identify reservoirs of resistance genes associated with relevant farm animals used for food production [6,7]. In the Republic of Kazakhstan, there is a lack of information on the antimicrobial resistance profiles of pathogens causing intestinal infections in calves; thus, there is an urgent need to monitor the use of antimicrobials on livestock farms. Monitoring antimicrobial resistance (AMR) among *E. coli* can provide information on the indiscriminate administration of antibacterial drugs to farm animals and the dynamics of transmission and development of resistance factors.

Considering the limited information on *E. coli* strains causing intestinal infections in calves on livestock farms in Kazakhstan, the aim of this work was to study the drug resistance profile and genome of *E. coli* isolated from the feces of animals with signs of escherichiosis. This is the first report on the AMR profile of an *E. coli* isolate obtained from a calf with evidence of escherichiosis in Kazakhstan. We present the genomic and drug resistance profiles of bovine multidrug-resistant *Escherichia coli* strain 35 isolated in Kazakhstan.

## 2. Materials and Methods

### 2.1. Sample Collection, Isolation, and Identification of E. coli

Samples were collected from the top layers (not touching the farm soil) of fresh feces and placed into sterile tubes (without any contact with or harm to animals). For bacterial isolation, 10 g of fecal sample was placed into 90 mL of buffered peptone water (HiMedia, Laboratories Pvt. Limited, Mumbai, India) and incubated at 37 °C for 12 h. The resulting non-selective enriched material was used for bacterial isolation. A loop-full of pre-enriched culture was inoculated onto Endo agar (TM Media, Delhi, India) to isolate *E. coli*, and then incubated at 37 °C for 24 h. Colonies typical for *E. coli* were selected based on morphological appearance—namely, those that were dark red with a metallic sheen. The presumably pure *E. coli* isolates were further analyzed using a Biolog system (Hayward, CA, USA) according to the manufacturer’s standard protocol. Briefly, purified *E. coli* cultures were inoculated onto BUG (Biolog Universal Growth agar, Hayward, CA, USA) agar medium, and then bacterial suspensions were prepared from the grown colonies at the appropriate density in inoculation fluid A (IF A, Hayward, CA, USA) according to the instructions. The prepared inoculums were added to the wells of a Biolog GEN III microplate and the plate was incubated for 22 h at 33 °C. After incubation, the optical density of the medium in each well of the microplate was measured on a plate spectrophotometer at a wavelength of 590 nm, using 750 nm as a reference. The difference in optical density readings between the experimental wells and the control wells was used to record the presence of a reaction (+), an intermediate state (+/−), or no reaction (−). The biochemical fingerprint obtained was entered into the Biolog Microlog 3.70 database (Biolog, Hayward, CA, USA), which showed the probability and similarity of the genus and species identity of the investigated bacterial culture [8].

The purified cultures of *E. coli* were then stored in 20% glycerol added to TSB broth at −80 °C.

### 2.2. DNA Extraction, Genome Sequencing, Assembly, and Annotation

For total DNA extraction, the bacterial culture was grown for 12 h in 5 mL of TSB (Condalab, Madrid, Spain) at 37 °C for 18–20 h. After that, the bacterial cells were precipitated by centrifugation at 6000 rpm for 30 min. The supernatant was discarded and the cells were resuspended in sterile phosphate-buffered saline and subjected to genomic DNA isolation using a DNeasy PowerLyzer Microbial Kit (QIAGEN, Hilden, Germany) according to the supplier’s instructions. The concentration of the isolated genomic DNA was assessed using a Qubit^®^ 3.0 fluorimeter and the Qubit dsDNA High Sensitivity Kit (Invitrogen, Carlsbad, CA, USA). The contamination with proteins and phenol was determined using Tecan’s NanoQuant Plate (Tecan, Männedorf, Switzerland) at the optical wavelengths of 260 and 280 nm.

Whole-genome sequencing of *E. coli* strain 35 was conducted on the Illumina Miseq platform in the paired-end mode; it had a read length of 2 × 300 bp (Miseq kit v3, Illumina, Cambridge, UK). The library was prepared using the Nextera XT DNA library preparation kit (Illumina, Cambridge, UK). Quality control of the short nucleotide reads was executed with FastQC v0.12.1 [9].

Trimmomatic 0.38.0 software was used to remove the adapter [10]. Sequences of low quality (<Q30) were removed, after which, the remaining reads were, on average, 50 to 250 bps long. The de novo assembly of the genome was carried out using SPAdes 3.12.0 software [11]. The quality of the assembled genome was determined by comparison with the reference genome using Geneious Prime, version 2023 [12]. The assembled genome was annotated using the NCBI Prokaryotic Genome Annotation Pipeline (PGAP), GeneMarkS-2+ [13], RAST [14], and Bacta. The OrthoANIu tool [15] was used to compare the prokaryotic genome sequence when classifying and identifying bacteria (https://www.ezbiocloud.net/tools/ani (assessed on 12 October 2024). The Comprehensive Antibiotic Resistance Database was used to detect resistance genes [16].

### 2.3. Antimicrobial Susceptibility Testing

Phenotypic antibiotic susceptibility testing (Condalab, Spain) and result interpretation were performed according to Comité de l’antibiogramme de la Société Française de Microbiologie, Recommandations Vétérinaires (CASFM Veterinary standard), and Clinical and Laboratory Standards Institute (CLSI) recommendations [17,18].

The following eight antibiotics were used in the experiment: beta-lactam (ampicillin (AMP-10), amoxicillin/clavulanic acid (AMC-30)), tetracyclines (tetracyclin (TET-30)), fluoroquinolones (enrofloxacin (ENR-5)), aminoglycosides (gentamicin (GEN-10)), sulfonamides (sulfamethoxazole/trimethoprim (SXT-25)), amphenicols (florfenicol (FLO-30)), and peptide antibiotic (colistin (COL-10)). The inoculum was prepared through the direct suspension of colonies from a pure 18–24 h bacterial culture in a sterile isotonic solution. The obtained inoculum, which corresponded to a density of 0.5 according to the McFarland turbidity standard, was inoculated into Petri dishes with pre-prepared Mueller–Hinton agar without additional additives. After that, disks with antibiotics from commercial kits were placed on the agar surface and incubated for 24 h at 37 °C, after which the diameter of the lysis zones was measured (Table 1). The susceptibility of the bacterial isolate to colistin was determined by the broth disk elution method. The experiment and interpretation of the obtained results were performed according to CLSI recommendations (no growth of bacterial culture at colistin concentration ≤ 1 µg/mL—sensitive, growth of bacterial culture at concentration ≤ 2 µg/mL—intermediate-resistant, growth of bacterial culture at concentration ≥ 4 µg/mL—resistant). Resistance to three or more classes of antibiotics was the criterion used to classify a bacterial isolate as a multidrug-resistant (MDR) isolate [19]. Tests were conducted in triplicate, with *E. coli* ATCC 25922 as the quality control strain.

## 3. Results

### 3.1. Overview of E. coli Strain 35 Draft Genome Assembly

**Figure 1:**
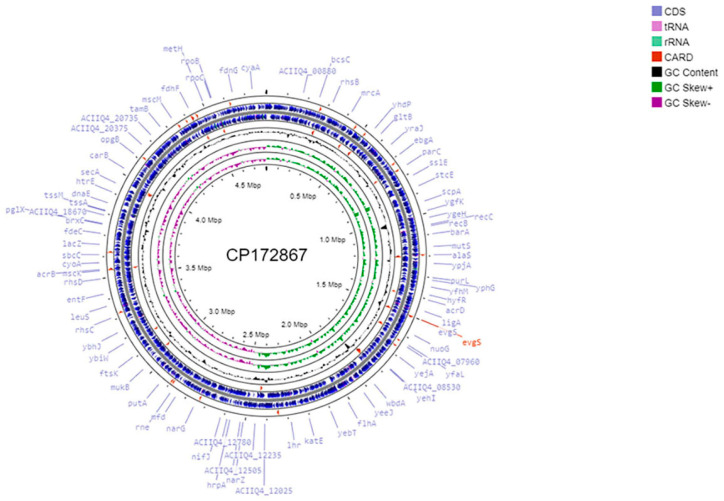
The genome map of *E. coli* strain 35 built using the CGView server (https://proksee.ca/ assessed on 8 November 2024). The blue arrows represent CDSs; green peaks represent GC-skew+; purple represents GC-skew-; and black peaks represent G+C content.

This work presents the draft genome sequence of multidrug-resistant *E. coli* strain 35 (Table 2, Figure 1). This strain was isolated from the feces of a diarrheic Holstein–Friesian calf on a cattle farm in Almaty oblast. The represented genome has a total length of 4,803,482 bp and contains 4986 genes, including 122 RNA genes.

The taxonomic affiliation of the strain was predicted by comparing the entire genome with OrthoANI values using the genome-to-genome distance calculator (GGDC). The most closely related microorganism found in the GenBank database was *Escherichia coli* strain CFS3246 (CP026929.2) (Table 3).

### 3.2. Functional Annotation of the Genome

**Figure 2:**
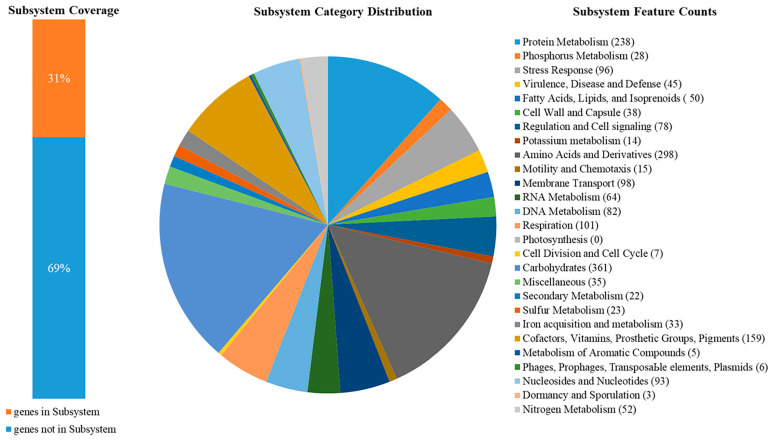
Subsystem statistics information on genome *E. coli* strain 35 obtained using RAST annotation. The subsystem categories and corresponding counts are presented in the legend.

Annotation in RAST enabled us to determine that 31% of the genes were functionally significant and represented 26 functional groups (Figure 2). The most common genes (more than 100) belonged to carbohydrates; amino acids and derivatives; protein metabolism; cofactors, vitamins, prosthetic groups, and pigments; and respiration, with 361, 298, 238, 159, and 101 genes in each group, respectively. The least common genes (fewer than 10) belonged to the following groups: cell division and cell cycle (7 genes); phages, prophages, transposable elements, and plasmids (6 genes); metabolism of aromatic compounds (5 genes); and dormancy and sporulation (3 genes). In the remaining 17 functional groups, the number of genes varied from 14 to 98.

### 3.3. Drug Resistance Profile

The results of the drug sensitivity tests for the investigated strain are presented in Table 4.

According to the results, the isolate under study demonstrated resistance to all eight antimicrobial drugs tested.

In addition, the presence of antibiotic resistance genes in the genome of *E. coli* strain 35 was determined using the Comprehensive Antibiotic Resistance Database (CARD; card.mcmaster.ca, accessed on 8 November 2024). The predicted resistance genes in the genome are presented in Table 5. The profile of predicted drug resistance genes included 45 efflux pumps, of which 21 belonged to the RND family, 16 to the major facilitator superfamily (MFS), 5 to the ATP-binding cassette (ABC) family, and 1 to the kdpDE two-component regulatory system for aminoglycoside resistance; additionally, 2 were represented by small multidrug resistance (SMR) proteins. Nine predicted drug resistance genes were involved in the mechanism of altering the target action of the antibiotic; one gene was involved in the enzymatic inactivation of the antibiotic (ampC-type beta-lactamase).

## 4. Discussion

The uniqueness of the geographical location and area of the Republic of Kazakhstan is the main reasons why animal husbandry was developed there; it accounts for up to 70 percent of agricultural land and provides employment for about 10 percent of the population. However, despite the measures implemented by the Government of Kazakhstan to develop animal husbandry, increasing its profitability and strengthening the country’s food security, infectious diseases in farm animals still pose a serious threat. One such disease is gastrointestinal infections in calves caused by pathogenic forms of *E. coli*. This disease is prevalent in many farms in Kazakhstan, and worldwide, and it is characterized by high contagiousness, severe diarrhea, and high mortality, resulting in significant economic losses [20]. Moreover, sick calves with escherichiosis are the main reservoir for the transmission of pathogenic *Escherichia coli* to humans, making the effective prevention and treatment of this disease an important challenge [21].

In addition, this problem is complicated by the global spread of antibiotic resistance among bacteria, which significantly reduces the effectiveness of antibiotic therapy. The Global Antimicrobial Resistance and Surveillance System (GLASS) 2022 report shows alarming rates of resistance among major bacterial pathogens. For example, the reported median rates in 76 countries indicate that among infections caused by *E. coli*, 42% are already resistant to third-generation cephalosporins [22]. The transmission of antibiotic resistance factors from animal pathogens to human pathogens poses risks. Therefore, continuously monitoring the spread of antibiotic resistance, especially among commensal bacteria such as *E. coli*, is necessary to model the future pathways of resistance and to take timely measures to prevent further spreading.

Research on antibiotic-resistant *E. coli* isolated from calves in the Republic of Kazakhstan has been carried out in very limited quantities. There are data on the contamination of dairy products with *E. coli* and its resistance to antimicrobial preparations. It was shown that *E. coli* contamination was found in 31.4% of commercial samples of Kazakhstani cheeses, and these isolates of *E. coli* showed resistance to 65% of tested antibacterial drugs and contained resistance genes β-lactams, sulfonamides, and quinolones [23]. Mendybayeva et al. showed the resistance of *E. coli* isolates (from biological material from animals and birds) to drugs such as β-lactams, fluoroquinolones, nitrofurans, and tetracyclines [24]. The lack of information on antimicrobial resistance in cattle in Kazakhstan prompted us to conduct this study. Therefore, the aim of this work was to study the drug resistance profile and genome of a bacterial strain isolated from a calf with signs of escherichiosis.

Whole-genome sequencing is increasingly being used to analyze the genetic profile of drug-resistant strains and the mechanisms of resistance gene transfer [25]. It has been shown that *E. coli* is able to develop antibiotic resistance through various mechanisms, including efflux pumps, biofilm formation, and the enzymatic modification of antibiotics [26,27]. According to the CARD prediction results, the *E. coli* strain under study has three resistance mechanisms, with the efflux pump being the predominant mechanism. In addition, to assess potential antimicrobial resistance factors, a study was performed to compare predicted genotypic resistance (the presence of resistance genes that cause antibiotic resistance) with phenotypic drug resistance data based on antimicrobial susceptibility testing.

Phenotypic resistance studies showed that the strain was resistant to drugs belonging to seven classes of antibiotics, including colistin. The emergence of *E. coli* strains resistant to critical antimicrobials such as colistin is a worldwide problem. The World Health Organization (WHO) has classified third- and higher-generation cephalosporins, quinolones, and colistin as a priority among critical antimicrobials for the treatment of serious infections caused by multidrug-resistant bacteria [28].

Detected resistance to beta-lactam antibiotics such as ampicillin and amoxicillin–clavunic acid, is provided by a group of enzymes—AmpC-type beta-lactamases (bla). The *ampC* gene encoding blaEC family class C beta-lactamase was found in the genome of *E. coli* strain 35. These are cephalosporinases that hydrolyze most penicillins, cephalosporins, oxime-cephalosporins, and monobactams. These drugs are among the mainstays in the treatment of bacterial infections, and pathogen resistance to their action is a serious clinical problem, exacerbated by the possibility of switching from inducible to continuous enzyme production during antibiotic administration; classical beta-lactamase inhibitors (clavulanic acid and sulbactam) have no effect [29].

In addition, the studied isolate showed resistance to trimethoprim/sulfamethoxazole. Usually, resistance to this antibiotic is due to the presence of *sul 1*, *sul 2*, and *sul 3* genes, while the carriage of *dfr A1*, *dfr A12*, *dfr A14*, and *dfrA17* genes is responsible for trimethoprim resistance, which has previously been described in studies of Egyptian isolates from calves and mastitis pathogens [30,31,32]. However, the above genes were not detected in the studied genome, and resistance to trimethoprim–sulfamethoxazole is most likely due to the presence of the *rsmA* gene. The investigated isolate’s resistance to colistin is probably related to the presence of the *eptA* gene and the *arnBCADTEF* operon in the genome. It was shown that chromosomal point mutations resulting in the constitutive expression of *eptA* and the *arnBCADTEF* operon lead to the modification of lipopolysaccharide through the addition of 4-amino-4-deoxy-l-arabinose (l-Ara4n) and/or phosphoethanolamine (pEtN) groups to lipid A, which leads to a decrease in the negative charge of the cell membrane and a decrease in antibiotic binding [33,34,35].

The isolation of *E. coli* strains resistant to many antibacterial drugs indirectly indicates that there is permanent uncontrolled use of antibiotics on the farm where the calves are kept. This significantly deteriorates the quality of the final product and creates a high risk of multidrug resistance among pathogenic microorganisms, which is dangerous for both veterinary and public health.

The functional annotation of the studied *E. coli* strain’s genome showed an abundance of genes not only related to metabolic processes and vitamin and amino acid biosynthesis, but also belonging to the category of “Virulence, Disease and Defense”, among them genes involved in adhesion, resistance to antibiotics, and the synthesis of bacteriocins and antibacterial peptides.

According to virulence profiles, *E. coli* is categorized into different pathotypes based on the presence of specific virulence genes [36], with common pathotypes associated with newborn calf diarrhea, including enteropathogenic (EPEC), shiga-toxin-producing (STEC), enterotoxigenic (ETEC), and enteroaggregating (EAEC) *E. coli* [37]. Although the *E. coli* isolate studied in this work was not tested for the presence of virulence markers of these pathotypes via PCR, genome annotation revealed virulence genes that are also found in pathogenic strains, for example, the *fimH* gene encoding type I fimbriae [38], which is associated with various pathotypes in both humans and animals, and the *hra* (heat-resistant agglutinin) gene encoding heat-resistant agglutinin, which is an additional factor in the colonization of enteroaggregative *Escherichia coli* (EAEC) strains [39]. The *yehABCD* genomic cluster encoding YehD fimbriae (YDF) was also discovered, which is associated with the virulence of EAEC strains carrying AAF/IV, contributes to the formation of biofilms, and improves the adhesive properties of the bacteria [40]. The presence of the *FdeC* gene encoding an intimin-like protein was identified, which has recently been shown to play a potential role in the colonization of *E. coli* in the terminal rectum of bovines [41].

This work revealed that the isolated *E. coli* strain possesses both pathogenicity factors and various mechanisms of antibiotic resistance. In addition, the data of phenotypic resistance of the isolated strain are confirmed by the presence in its genome of genes responsible for antibiotic resistance factors. It is assumed that the acquisition of antibiotic resistance may occur in accordance with the pre-existence of a certain genetic profile, including genes encoding virulence factors such as *ompA*, *malX*, and *hlyA*. For example, in uropathogenic *E. coli* isolates, it was shown that some surface adhesions and an iron-acquisition system are associated with antibiotic susceptibility. In contrast, *ompA* and some other genes associated with pathogenicity factors are predominantly associated with antibiotic resistance [42].

Many studies have reported that the increased expression of various virulence factors causes microbes to have increased pathogenicity. It was also found that the expression of antibiotic resistance genes increases the pathogenicity of microorganisms. Thus, antibiotic resistance genes are considered a virulence factor. The ability to form biofilms and the presence of pili, fimbriae, and flagella contribute not only to the adhesion of microbes to biotic or abiotic surfaces, but also to the expression of “quorum sensing” signals, which leads to increased pathogenicity and antibiotic resistance. Another unfavorable factor for antibiotic therapy is the selection of hybrid plasmids (carrying different pathogenicity factors in association with drug resistance genes) by antibiotics, resulting in the selection of virulence factors [43,44,45,46].

This study did not investigate the relationship between the antibiotic resistance profile and virulence genes, as additional studies with a large number of bacterial isolates are needed to obtain such results. However, the preliminary results indicate multidrug resistance in the investigated strain, suggesting the indiscriminate use of antibiotics in this livestock, which causes selective pressure favoring the emergence of resistant strains. These results call for the rational use of antimicrobials on livestock farms; the results can also be used to make recommendations to the government to regulate antimicrobials used in veterinary medicine.

## 5. Conclusions

This study provides preliminary information on the antibiotic resistance of *Escherichia coli* isolated from the feces of a sick calf in Kazakhstan. The obtained data indicate the need for rational antibiotic use and surveillance of antimicrobial resistance among farm animals. Future work, which will include the most significant number of isolates in the territory of the Republic of Kazakhstan, will contribute to a better understanding of the relationship between the genome structure of isolates and their drug resistance profiles and diseases, as well as the use of antimicrobials. Our results confirm previous reports of multidrug-resistant *E. coli* from calves carrying different resistance genes that are a significant factor in the resistance to clinically important drugs. Regular surveillance of the antibiotic resistance profiles of bacterial pathogens isolated from animals by phenotypic or genotypic testing is crucial to determine the emergence of antimicrobial resistance and the associated risk to humans.

## Figures and Tables

**Table 1:** Interpretation breakpoints of the various antimicrobial agents used in the study.

Antibiotic	Concentration (µg)	Breakpoint (mm)	
		Sensitive (S)	Resistant (R)
Tetracyclin (TET) *	30	≥19	<17
Gentamicin (GEN)	10	≥18	<16
Sulfamethoxazole/trimethoprim (SXT)	25	≥16	<10
Enrofloxacin (ENR)	5	≥19	<19
Amoxicillin/clavulanic acid (AMC)	30	≥21	<14
Florfenicol (FLO)	30	≥19	<19
Ampicillin (AMP) **	10	≥17	≤13

* Breakpoint indicated according to CASFM, 2021. ** Breakpoint indicated according to CLSI, 2023.

**Table 2:** Genome characteristics of *E. coli* strain 35.

Genome size (bp)	4,803,482
GC% content	50.9%
Number of contigs ≥ 1000 bp	241
Max length of contig	180,322
N50 length (bp)	38,594
Genes (total)	4986
CDSs (total)	4864
Genes (RNA)	122
tRNAs	88

**Table 3:** Results of whole genome comparison of *E. coli* strain 35.

Strain	Accession	Size (bp)	GC%	OrthoANI Value (%)	GGDC Distance
*Escherichia coli strain CFS3246*	*CP026929.2*	4,803,482	50.87	99.88	0.0017
*Escherichia coli strain E275 a*	*CP035865.1*	5,044,130	50.74	99.13	0.0855
*Escherichia coli strain 17MR471*	*CP051158.1*	4,765,524	50.71	99.22	0.0875
*Escherichia coli O19H7 strain 730V1*	*CP061764.1*	5,113,454	50.95	98.59	0.1462
*Escherichia coli strain 1283*	*CP023371.1*	4,677,088	50.79	99.68	0.0569
*Escherichia coli strain DA33133*	*CP029574.1*	4,771,844	50.82	99.58	0.0639
*Escherichia coli strain KSE-B05*	*CP125003.1*	4,710,376	50.80	99.60	0.0613
*Escherichia coli strain KFS-D01*	*CP125039.1*	4,710,765	50.81	99.61	0.0573
*Escherichia coli strain ABW_A32*	*CP067303*	4,602,727	50.69	99.75	0.0514
*Escherichia coli strain ABW_A33*	*CP067299*	4,625,685	50.65	99.77	0.0535

**Table 4:** Results of *E. coli* drug susceptibility testing.

Strain	Antibiotics (µg)							
	Ampicillin (AMP-10)	Tetracyclin (TET-30)	Gentamicin (GEN-10)	Sulfamethoxazole/trimethoprim (SXT-25)	Enrofloxacin (ENR-5)	Colistin (COL-10)	Amoxicillin/clavulanic acid (AMC-30)	Florfenicol (FLO-30)
*E. coli* strain 35	R *	R	R	R	R	I	R	R

* R: resistance, I: intermediate.

**Table 5:** Genes associated with the antibiotic resistance of the *E. coli* strain 35.

Resistance Mechanism	Antimicrobial Resistance Gene Family	Genes	Drug Class
Antibiotic efflux	MFS antibiotic efflux pump	emrB, emrA, emrR, emrK, emrY, mdtH, mdtG, Escherichia coli mdfA, leuO, mdtM, mdtN, mdtO, mdtP, evgS, evgA, H-NS	Aminoglycoside antibiotic, fluoroquinolone antibiotic, tetracycline antibiotic, phosphonic acid antibiotic, lincosamide antibiotic; nucleoside antibiotic; phenicol antibiotic; penam; macrolide antibiotic; cephalosporin; cephamycin; disinfecting agents and antiseptics
	RND antibiotic efflux pump	gadX, gadW, mdtF, mdtE, CRP, AcrF, AcrE, AcrS, rsmA, acrD, baeR, baeS, mdtC, mdtB, mdtA, Escherichia coli acrA, acrB, cpxA, Escherichia coli AcrAB-TolC with MarR, Escherichia coli AcrAB-TolC with AcrR, marA	Aminoglycoside antibiotic; aminocoumarin antibiotic; fluoroquinolone antibiotic; cephalosporin; glycylcycline; penam; tetracycline antibiotic; rifamycin antibiotic; phenicol antibiotic; disinfecting agents and antiseptics
	SMR antibiotic efflux pump	KpnE, KpnF	Macrolide antibiotic; aminoglycoside antibiotic; cephalosporin; tetracycline antibiotic; peptide antibiotic; rifamycin antibiotic; disinfecting agents and antiseptics
	kdpDE two-component regulatory system	kdpDE	Aminoglycoside antibiotic
	ABC antibiotic efflux pump	YojI, msbA, TolC, soxR, soxS	Peptide antibiotic; nitroimidazole antibiotic; carbapenem; cephalosporin; glycylcycline; cephamycin; rifamycin antibiotic; phenicol antibiotic; penem; monobactam; carbapenem; cephalosporin
Antibiotic target alteration	Elfamycin-resistant Fluoroquinolone-resistant Fluoroquinolone-resistant Glycopeptide resistance gene cluster; Van ligase Penicillin-binding protein mutations Pmr phosphoethanolamine transferase Undecaprenyl pyrophosphate-related proteins	EF-Tu, gyrA, parC, vanG, PBP3, ArnT, PmrF, eptA, bacA	Elfamycin antibiotic; fluoroquinolone antibiotic; glycopeptide antibiotic; cephalosporin; cephamycin; penam; peptide antibiotic
antibiotic inactivation	ampC beta-lactamase	Escherichia coli ampC-type beta-lactamase	Cephalosporin; penam

## Data Availability

The raw genome sequencing data of Illumina MiSeq are deposited in NCBI in FASTQ format with BioSample: SAMN44529025, under BioProject PRJNA1180887. The assembled genome is available in NCBI GeneBank under CP172867.1.
